# *Chrysothrix* sp., a Lichen from Paposo Fog Oasis: Antibacterial Potential to Combat Multidrug-Resistant ESKAPE-E Pathogens

**DOI:** 10.3390/microorganisms14081766

**Published:** 2026-08-11

**Authors:** Martín I. Escalona-Acuña, Ángel Dzul-Beh, Silvia Tapia, Pedro Cortés Peña, Ivan Brito, Jorge Bórquez, Gloria María Molina Salinas, Patricio R. Orrego

**Affiliations:** 1 Departamento de Química, Facultad de Ciencias Básicas, Universidad de Antofagasta, Antofagasta 1270300, Chile; martin.escalona.acuna@ua.cl (M.I.E.-A.); pedro.cortes.pena@uantof.cl (P.C.P.); ivan.brito@uantof.cl (I.B.); jorge.borquez@uantof.cl (J.B.); 2 Unidad de Investigación Médica Yucatán, Instituto Mexicano del Seguro Social, Merida 97150, Yucatan, Mexico; angeldzulbeh1992@gmail.com; 3 Departamento Biomédico, Facultad de Ciencias de la Salud, Universidad de Antofagasta, Antofagasta 1270300, Chile; silvia.tapia@uantof.cl

**Keywords:** antibacterial, calycin, *Chrysothrix* sp., ESKAPE-E group, fog oasis, methanolic extract, secondary metabolites

## Abstract

Lichens are mutualistic symbiosis between a fungus (mycobiont) and an alga or cyanobacteria (photobiont), forming metabolically versatile holobionts capable of producing diverse secondary metabolites that facilitate their survival in extreme environments. *Chrysothrix* species, commonly known as “gold dust lichens,” are characterized by their vivid yellow thalli and their production of pulvinic acid derivatives, although their bioactive potential remains poorly explored. In this study, using organic chemistry techniques, we characterized the methanolic extract of *Chrysothrix* sp.—collected from the Paposo Fog Oasis in northern Chile, a unique coastal ecosystem sustained by persistent fog (“camanchacas”) within the Atacama Desert—and identified calycin as its major secondary metabolite through chromatographic purification and single-crystal X-ray diffraction. Antibacterial assays revealed that both the crude methanolic extract and purified calycin exhibited selective inhibitory activity against multidrug-resistant Gram-positive ESKAPE-E pathogens. Minimum Inhibitory Concentrations (MICs) ranged from 125 to 500 μg/mL, with the strongest effects observed against *Enterococcus faecium* (MDR, VRE) and *Staphylococcus aureus* (MDR, MRSA). No meaningful activity was detected against Gram-negative bacteria, consistent with the known permeability barrier conferred by the outer membrane. This work provides the first evidence of antibacterial activity for calycin isolated from *Chrysothrix* sp., highlighting the relevance of pulvinic acid derivatives as promising scaffolds for antimicrobial development. These findings highlight the potential of lichen-derived agents and alternative sources of antibacterial agents to address the global challenge of antimicrobial resistance.

## 1. Introduction

Lichens are defined as a symbiosis between a mycobiont (fungal partner) and a photobiont (photoautotrophic partner), forming a complex microbial consortium that constitutes a largely unexplored source of bioactive secondary metabolites [1]. Currently, 19,387 accepted species have been described, comprising 995 genera, 115 families, 39 orders, and eight classes [2]. Their ability to colonize nearly all terrestrial habitats—from tropical to polar regions—reflects the success of this symbiosis. Owing to their metabolic versatility, lichens can withstand extreme temperature fluctuations and high UV radiation [3]. This mutualistic interaction gives rise to lichen thalli with distinct morphology, anatomy, physiology, genetics, and ecology, forming complex holobionts composed of obligate symbionts [4]. Based on thallus morphology and substrate, lichens are classified as crustose, foliose, and fruticose [5].

The production of secondary metabolites in lichens is highly complex and strongly influenced by environmental factors such as light intensity, UV exposure, altitude, temperature variations, and seasonality [6]. Many lichens-derived compounds have demonstrated significant pharmaceutical potential [7,8] and continue to attract interest as alternative therapeutics agents worldwide [9]. These metabolites exhibit antimicrobial, antioxidant, anti-inflammatory, cytotoxic, analgesic, antipyretic, and antiviral activities, positioning lichens as promising sources of pharmaceutically relevant natural products [6].

Interest in lichen secondary metabolites has intensified due to the global antimicrobial resistance (AMR), defined as the ability of microorganisms to survive and proliferate despite exposure to antimicrobial agents. Increased antibiotics use worldwide has accelerated the emergence of resistant strains and associated clinical complications [10]. In 2021, bacterial resistance was associated with 4.71 million deaths, including 1.14 million deaths attributable to multidrug-resistant bacteria [11]. Projections estimated that AMR could cause 10 million deaths annually by 2050 [12].

The most critical bacteria known for their ability to evade antibiotics action are grouped under the acronym ESKAPE-E (*Enterococcus faecium*, *Staphylococcus aureus*, *Klebsiella pneumoniae*, *Acinetobacter baumannii*, *Pseudomonas aeruginosa*, *Enterobacter* spp., and *Escherichia coli*) [13]. The World Health Organization (WHO) has further classified major bacterial pathogens into critical, high-priority, and medium-priority categories, to guide the development of new antibacterial agents [14]. Despite its exceptional biodiversity, research on lichens in Chile remains limited, even though the country is considered one of the global hotspots for lichen diversity [15].

The lichen genus *Chrysothrix*, commonly known as ‘gold dust lichens’, is characterized by its bright yellow crustose thalli and the production of pulvinic acid derivates, terpenes, depsides, and other diagnostic metabolites. Although widely distributed across continents and substrate, its biological activities (particularly antibacterial properties) remain poorly documented.

This study investigated the chemical composition and antimicrobial potential of *Chrysothrix* sp., evaluating its activity against multidrug-resistant ESKAPE-E pathogens. Our specimen, collected in the Paposo Fog Oasis in northern Chile, allowed us to assess its potential as a source of new bioactive compounds to address the global challenge of antimicrobial resistance.

## 2. Materials and Methods

### 2.1. Sample Collection and Morphological Characterization

*Chrysothrix* sp. was collected at approximately 742 m above sea level (masl) from the spines of a columnar cactus morphological similar to *Eulychnia breviflora*. Samples were carefully detached using a sterile metal laboratory spatula and transferred into 50 mL conical-bottom polypropylene tubes. The specimen was identified based on macroscopic morphology (bright lemon-yellow crustose thallus, indeterminate margins, compact surface) following regional taxonomic keys [16].

The material was stored at room temperature in the dark until processing. Photographs of specimens were taken using a Samsung Galaxy S23 Ultra smartphone and the geographic coordinates were recorded with the GPS Logger application (25°00′39.80″ S 70°27′06.01″ W) (Figure S1).

### 2.2. Extraction and Purification of Secondary Metabolites

Lichen samples of *Chrysothrix* sp. (2.8 g) were air-dried at room temperature for 48 h and subsequently macerated in methanol (p.a. 1.06009.2500) for 5 days at room temperature. The extract was then filtered, yielding 125 mL of crude methanolic extract. The extract was concentrated under reduced pressure (40 °C, 337 mbar) using a rotary evaporator, yielding 1.2 g of crude extract. This yield is consistent with the high content of methanol-soluble pigments characteristic of *Chrysothrix* species.

The crude extract (1.2 g) was subjected to a medium-pressure column chromatography using a 2.5 cm × 48 cm column (Aceglass inc, Vineland, NY, USA) packed with silica gel (Kieselgel 60 H, 55 µm particle size, Merck, Darmstadt, Germany). An isocratic mobile phase of n-hexane: ethyl acetate (80:20 *v*/*v*) was delivered at 10 mL-min using a FMI lab pump (Syosset, NY, USA). A total of 100 fractions (10 mL each) were collected and pooled based on thin layer chromatography (TLC) profiles (Kieselgel F254 plates, developed with n-Hexane: ethyl acetate 80:20 *v*/*v* ratio in 5 mL). The mixture was allowed to stand for 2 min so that the mobile phase could advance by capillary action along the chromatographic plate and separate the bands in the mixture.

### 2.3. Structural Elucidation via X-Rays Diffraction (XRD)

We analyzed 100 fractions using thin-layer chromatography (TLC), in which a pure compound was observed in fractions 41 through 44, which were then combined; we then waited two days for the solvent (hexane/ethyl acetate) to evaporate at room temperature, resulting in the spontaneous formation of a single crystal.

Single-crystal X-ray diffraction data were collected at 295 (2) K on a Bruker D8 Venture diffractometer equipped with a Photon-III C14 detector, using graphite monochromated MoKα (λ = 0.71073 Å) and Cu-Kα (λ = 1.54178 Å) radiation (Bruker Co.; Billerica, MA, USA). Diffraction frames were integrated with the APEX4 software package (Bruker, 2026) and were corrected for absorptions with SADABS [17] (Table S1).

The structures was solved by intrinsic phasing [18] using the OLEX2 software (version 15.0) [19] and refined with full-matrix least-squares methods based on F^2^ (SHELXL) [18]. All the non-hydrogen atoms were refined anisotropically. Hydrogen atoms were placed in calculated positions and refined riding models with isotropic displacement parameters. The structure was refined as merohedral using the twin law defined by the matrix [−1.0, 0; 0, 0.0; 0, 0–1]. Geometrical calculations were performed using the Platon program [20].

### 2.4. Assessment of Antibacterial Activity

#### 2.4.1. Reference Strains

The pathogenic bacteria used to evaluate the antimicrobial activity of the lichen extract were: *Enterococcus faecium* (ATCC 700221), *Staphylococcus aureus* (ATCC 29213, ATCC 43300), *Klebsiella pneumoniae* (ATCC 2146), *Acinetobacter baumannii* (ATCC 1605), *Pseudomonas aeruginosa* (ATCC 35032), *Escherichia coli* (ATCC 25922; ATCC 35218) (Table S2).

#### 2.4.2. Agar Disk Diffusion Assay

A preliminary agar diffusion assay was performed using strains of *Staphylococcus aureus* (ATCC 29213) and *Escherichia. coli* (ATCC 25922) following Garcia et al. (2013) [21] with minor modifications.

Each strain was inoculated into 3 mL of Luria Bertain (LB) broth and incubated at 37 °C with shaking (200 rpm) for 16–18 h, as recommended for optical growth. Cultures were adjusted to an OD_600_ of 0.3, and 100 μL ofeach suspension were mixed with 9.9 mL of 0.8% LB agar and poured into 90 × 15 mm^2^ Petri dishes. After solidification, 6 mm-diameter filter paper disks (CL-501) previously impregnated with 5 μL methanolic extract (ME) or purified metabolite (PM) at concentrations of 0.25, 0.5, 1.0, and 2.0 mg/mL were placed on the agar surface. Ampicillin (30 μg/) served as the positive control, and methanol (p.a. 1.06009.2500) as the negative control. Plates were incubated at 37 °C for 16–18 h, and the antibacterial activity was assessed by the measuring inhibition zone. Halo values < 6 mm (disk diameter) were recorded as 0 mm. Images of the bioassays were recorded using a Samsung Galaxy S23 Ultra smartphone.

#### 2.4.3. Determination of the Minimal Inhibitory Concentration (MIC)

Determination of the MIC for ME and PM against the pathogens were performed using the resazurin microtiter assay (REMA) as described by Uc-Cachón et al. (2021) [22].

Bacteria were first grown on Muller–Hinton agar (MHA; Becton Dickinson, Franklin Lakes, NY, USA), and two to three colonies were suspended in 3 mL of Muller–Hinton broth (MHB). Cultures were incubated at 37 °C for 2–4 h until reaching turbidity level equivalent to the 0.5 McFarland standard (DEN-1; Biosan, Riga, Latvia). The resulting suspension was diluted 1:50 to obtain the working inoculum, of 10^6^ CFU/mL, 100 μL of this inoculum were mixed with 100 μL of extract solutions prepared at serial dilutions (1000 to 7.81 μg/mL) in sterile 96-well microplates (Corning Costar). Each assay included a positive control (antibiotic), a negative control (bacteria without extract), and a sterility control (broth).

Then microplates were incubated at 37 °C for 16 h. Them 30 μL of resazurin (Sigma, St. Louis, MO, USA) were added followed by 2 h incubation. The MIC was defined as the lowest concentration of the sample that prevented the color change from blue to pink. All assays were performed in duplicate [22].

#### 2.4.4. Determination of Minimal Bactericidal Concentration (MBC)

The MBC of ME and PM was determined by following Molina-Salinas et al. (2006) [23] with minor modifications. Briefly, 5 μL of the bacterial suspension exposed to the ME or PM at MIC, 2× and ½ MIC were taken directly from in the 96-well microplates and streaked onto MHB agar plates. The plates were incubated at 37 °C for 16 h. The MBC was defined as the lowest concentration at which no visible colonies were observed, indicating complete loss of bacterial viability.

### 2.5. Statistical Analysis

The results agar disk diffusion assay was presented as means ± standard deviations (mean ± SD). Data was analyzed using Microsoft Excel. Differences were considered significant at a *p*-value of less than 0.05. (*p* < 0.05).

## 3. Results

### 3.1. External Morphological Description of the Lichen Under Study and Its Geographic Location

**Figure 1:**
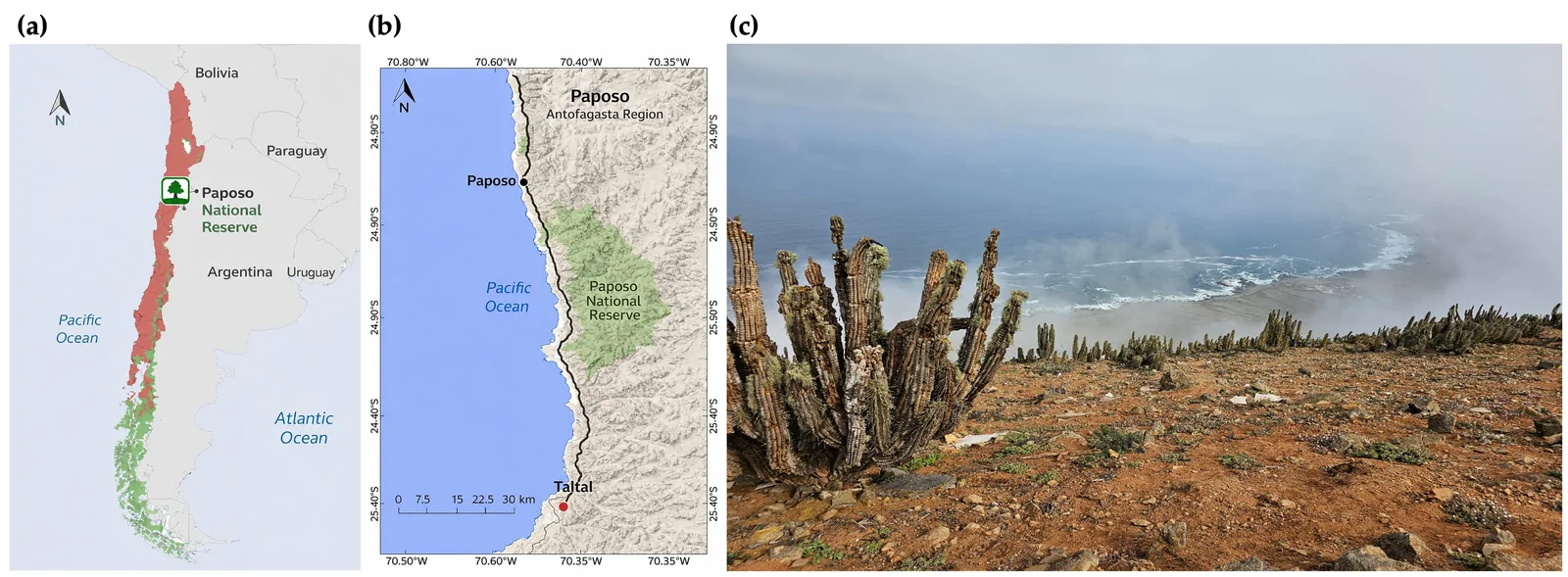
Geographic location of the study area in northern Chile. (**a**) Political map of Chile showing its national boundaries and highlighting the location of the Paposo National Reserve. (**b**) Geographic distribution of the Paposo National Reserve within the Antofagasta Region, depicted in green to indicate the protected area. (**c**) The camanchaca phenomenon, illustrating the persistent coastal fog characteristic of Atacama Desert and its occurrence fog over the Paposo Nature Reserve.

**Figure 2:**
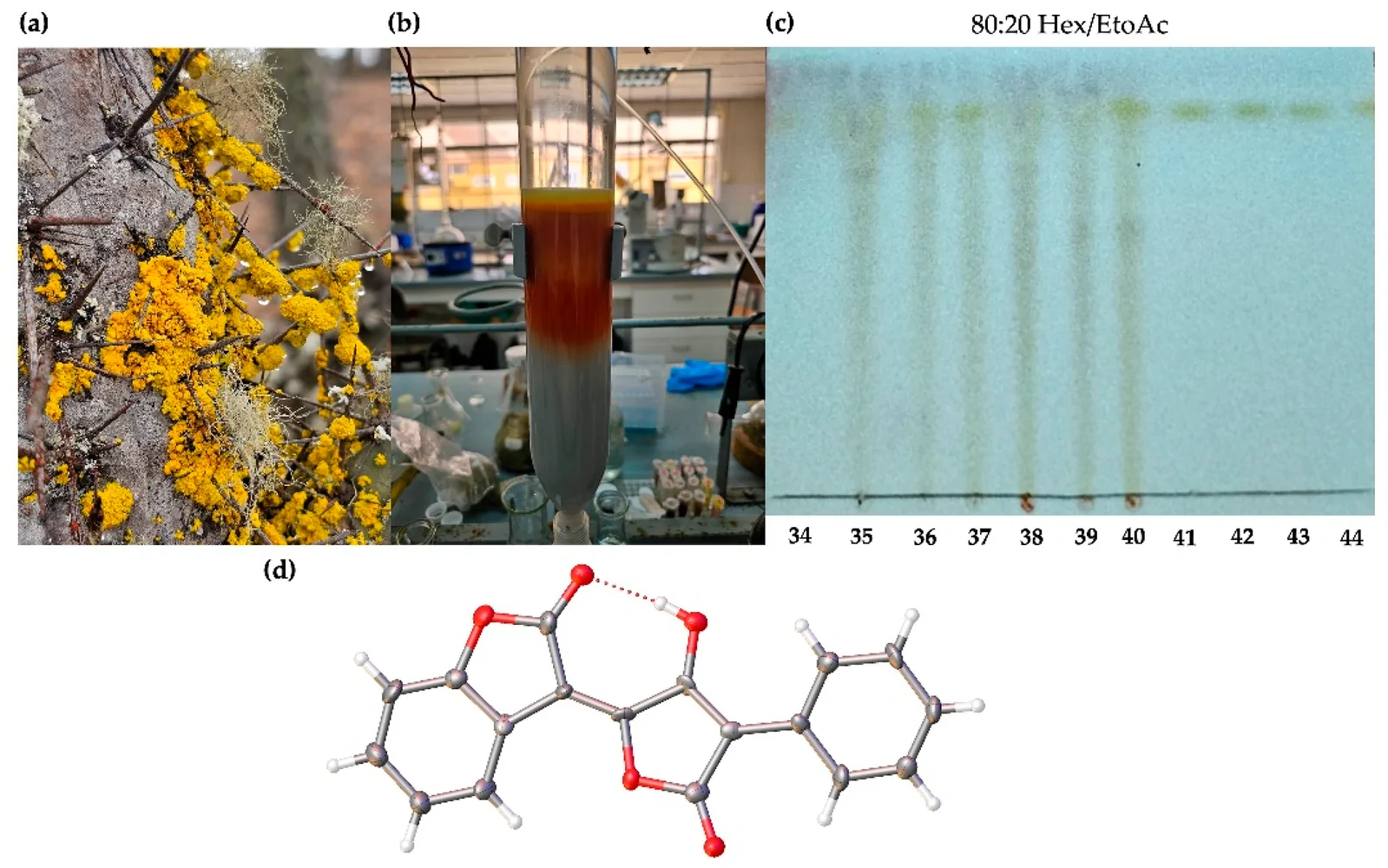
Morphology and chemical characterization of the major secondary metabolite methanolic extract of *Chrysothrix* sp. (**a**) External morphology of *Chrysothrix* sp., thallus. (**b**) Fractionation profile of the methanolic extract obtained by medium-pressure column chromatography using an 80:20 *v*/*v* n-hexane: ethyl acetate system. (**c**) Thin-layer chromatography of selected fractions obtained in (**b**), showing the dominant band corresponding to calycin (Rf = 0.62). (**d**) Chemical structure of the purified secondary metabolite identified as calycin.

The lichen specimens were collected in the Paposo Fog Oasis, a fog-dependent ecosystem characterized by a persistent coastal fog (camanchaca) occurring between 300 and 800 masl (Figure 1). The lichen under study exhibited a bright lemon-yellow crustose thallus firmly attached to the cactus spine, with individual thalli approximately 0.5–2.0 cm in length. The thallus surface was continuous and compact, and no visible reproductive structures were observed (Figure 2a).

### 3.2. Chromatographic Isolation and X-Ray Structural Determination of the Major Secondary Metabolite

The medium-pressure chromatography yielded 100 fractions, among which fractions 41, 42, 43 and 44 exhibited the highest degree of purity (8.4 mg), as confirmed by TLC analysis (Figure 2b,c). These fractions showed a single dominant TLC band (Rf = 0.62), corresponding to the major metabolite later identified as calycin. Structural elucidation of the PM was carried out by single-crystal X-ray diffraction. The molecular and supramolecular structures obtained from the PM of *Chrysothrix* sp. were identical to those previously reported by Fernandes et al. (2015) [24], confirming the identity of the isolated secondary metabolite as calycin (Figure 2d).

### 3.3. Antibacterial Activity

#### 3.3.1. Agar Disk Diffusion Assay

**Figure 3:**
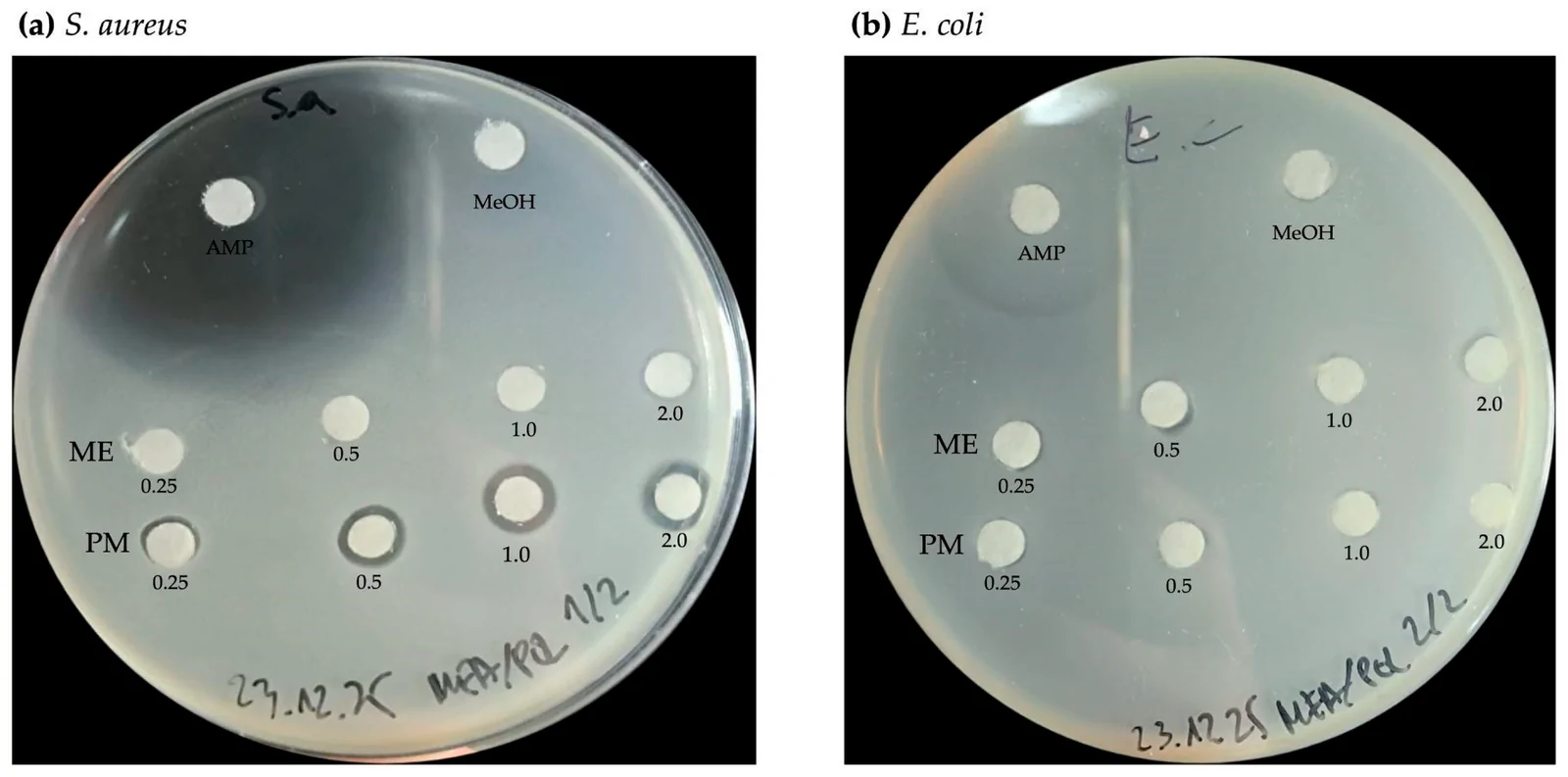
Disk diffusion assay evaluating the antibacterial activity of methanolic extract (ME)—and purified metabolite (PM) from *Chrysothrix* sp.—against bacterial pathogens. Inhibition zones produced by ME and PM are shown against *Staphylococcus aureus* (**a**) and *Escherichia coli* (**b**). Concentrations applied to each disk are expressed in mg/mL.

The evaluation of antimicrobial activity assessed through the modified disk diffusion assay showed a clear inhibitory effect against *S. aureus* (ATCC 29213), which was greater with the PM than with the ME. No inhibition zones were observed against *E. coli*, with halo diameters equal to the disk diameter (6 mm), recorded as 0 mm according to CLSI guidelines (ATCC 25922) (Figure 3, Table 1).

#### 3.3.2. Minimal Inhibitory Concentration

The antibacterial activity results obtained through MIC and MBC assays for the methanolic extract and calycin from *Chrysothrix* sp. are shown in Table 2.

## 4. Discussion

The study provides the first evidence of antibacterial activity for calycin and characterizes the chemical composition and antimicrobial activity of methanolic extract from the lichen *Chrysothrix* sp., native to the Paposo National Reserve, found growing epiphytically on a columnar cactus morphologically like *Eulychnia breviflora* [25]; a common substrate for lichen colonization [26]. The specimen was collected in the coastal escarpment of the Antofagasta Region in northern Chile, a unique ecosystem sustained entirely by persistent coastal fog (“camanchaca”) [27], within the Atacama Desert, the driest desert in the world [28].

Several species of the genus *Chrysothrix* have been reported in Chile, including *Chrysothrix pavoni*, during the 1997–1998 ENSO (El Niño-Southern Oscillation) event in the Tarapacá Region [29] and *C. candelaris*, *C. granulosa*, *C. pavonii*, and *C. chilensis* in the Coquimbo region [30]. Reported altitudinal ranges for *C. granulosa* and *C. pavonii* are 700 masl (Austral) and 450–700 masl (Neoaustral), respectively [31]. Our sample was collected at 740 masl, consistent with the altitudinal distribution and supporting morphological affinity with *C. granulosa* or *C. pavonii*.

Natural sources have demonstrated significant antimicrobial potential against ESKAPE-E group, such as the potent antibacterial activity of the n-hexane extract from the bark of *Matayba oppositifolia*, a plant used in traditional Mayan medicine [32], and similar activity has been reported for lichen extracts [33,34].

Among solvents used for lichen extraction, methanol, ethanol, ethyl acetate, and acetone are the most common, with methanol and acetone generally yielding the strongest antimicrobial activity due to their solubilizing properties [35,36]. In contrast, aqueous extracts rarely show activity, as reported for *Ramalina sinensis* and *Usnea barbata* [37], likely because many lichen secondary metabolites are poorly soluble in water or extracted in insufficient amounts. Alternative strategies include aqueous extraction followed by sterile filtration and freeze-drying to improve extraction yield and biological activity [38]. Recent comparative studies evaluating accelerated solvent extraction (ASE), maceration and natural deep eutectic solvents demonstrated that ASE with acetone is the most efficient method for extracting lichen acids physodalic, physodic, atranorin, and chloroatranorin [39].

In this study, methanol was selected due to its high polarity and broad solubilizing capacity, making it suitable for extracting phenolic compounds, lipids and fatty acids, anthocyanins, terpenoids, lignans, polysaccharides, proteins, and amino acids [6,40]. Rotary evaporation was chosen as a drying method because it preserves bioactive compounds while being faster and more cost-effective than freeze-drying [39].

More than 800 unique secondary metabolites have been identified in lichens, including depsides, depsidones, depsones, dibenzofurans, usnic acid derivatives, terpenoids, xanthones, chromones, and quinones, all exhibiting remarkable structural diversity [41]. Given the urgent need for new antimicrobial agents to combat multidrug-resistant pathogens [42], particularly methicillin-resistant *S. aureus* (MRSA), extended-spectrum β-lactamase-producing *Enterobacteriaceae* (ESβL), and carbapenem-resistant *Enterobacteriaceae* (CRE) [11], our work focused on members of the ESKAPE-E group, namely Enterococcus *faecium*, *Staphylococcus aureus*, *Klebsiella pneumoniae*, *Acinetobacter baumannii*, *Pseudomonas aeruginosa*, *Enterobacter* spp., and *Escherichia coli*. Despite the development of new antibiotics, and adjuvants such as latest-generation β-lactamase inhibitors, these pathogens continue to pose major therapeutic challenges [43].

Studies of methanolic extracts from Nepalese lichens collected between 60 and 8.848 masl reported antibacterial activity against *Bacillus subtilis* (including *Parmoterma* sp., *Heterodermia* sp., *Coccocarpia* sp., *Peltigera* sp., *Bulbolthrix* sp., *Canoparmelia* sp., *Ramalina* sp., *Everniastium* sp., *Cladonia* sp., and *Usnea* sp.) with only a subset of species showing activity against *S. aureus* (*Parmoterma* sp., *Canoparmelia* sp., *Ramalina* sp., *Everniastium* sp., *Cladonia* sp., and *Usnea* sp.), MIC 18.2–89.2 μg/mL [44]. No activity was observed against Gram-negative bacteria (*E. coli*, and *K. pneumoniae)*, or the opportunistic fungus *Candida albicans* [45]. Similar patterns have been reported for acetone and methanol extracts from *Usnea rubrotincta* and *Ramalina dumeticola*, which showed strong activity against Gram-positive bacteria but weak or no activity against Gram-negative species [46]. Among isolated compounds from lichens, usnic acid, barbatic acid atranorin exhibited the strongest activity against *S. aureus* [46], consistent with previous reports [47,48,49] and those isolated from *R. dumeticola*—atranorin (acetone extract), hyperhomosekikaic acid (acetone extract), and sekikaic acid (acetone extract)—all showed activity against *B. subtilis*; however, against *S. aureus*, only the compounds usnic acid (MIC 7.81 μg/mL), barbatic acid (MIC 62.5 μg/mL), and atranorin (MIC 62.5 μg/mL) exhibited activity [46]. Among the compounds studied, usnic acid was most effective against Gram-positive bacteria; this compound has previously been reported to possess such potential, as has atranorin [47,48,49]; whereas, the compounds 8-hydroxybarbatic acid from *U. rubrotincta* and hyperhomosekikaic acid from *R. dumeticola* exhibited activity against *B. subtilis*—the first time such activity has been described for these compounds—but they had no effect against *S. aureus* [46].

A systematic review of the past decade of lichen antimicrobial research [37] identified numerous genera with activity against the ESKAPE-E group, including *Usnea* sp., *Allocetraria* sp., *Evernia* sp., *Everniastrum* sp., *Parmotrema* sp., *Flavocetraria* sp., *Ramalina* sp., *Roccella* sp., *Niebla* sp., *Cladonia* sp., *Xanthoria* sp., *Heterodermia* sp., *Lobaria* sp., *Rhizoplaca* sp., *Tamnolia* sp., *Parmelia* sp., *Bulbothrix* sp., *Cetraria* sp., *Pseudovernia* sp., *Hypogymnea* sp., *Crypthothecia* sp., *Physcia* sp., *Stereocaulon* sp., *Phaeographis* sp., and *Trypethelevirens* sp.,. The most common solvents used were acetone and methanol. Notable MIC values include: *Xanthoria parietina* acetone extract against *E. faecium* (MIC = 15.6 μg/mL) [50]; *Cladonia uncialis* cetone extract against *S. aureus* (MIC = 0.5 μg/mL) [51], and *Xanthoria plitti* methanolic extract against *E. coli* (MIC = 10.0 μg/mL).

In our study, the methanolic extract and pure metabolite calycin of *Chrysothrix* sp. showed the strongest inhibitory effect against *E. faecium* MDR and VRE, with an MIC of 250 μg/mL and 125 μg/mL, respectively, followed by *S. aureus* MDR and MRSA (MIC = of 500 and 250 μg/mL). No significant activity was observed against Gram-negative bacteria (*K. pneumoniae*, *P. aeruginosa*, *A. baumannii* and *E. coli*), consistent with the requirement of MIC > 1000 μg/mL for meaningful inhibition. The moderate antibacterial activity observed for both the methanolic extract and calycin, compared with the reference antibiotics, is likely related to the intrinsic potency of pulvinic acid derivatives and the crude nature of the extract. Calycin is not primarily a bactericidal metabolite, and its activity is further limited by its inability to cross the outer lipopolysaccharide membrane of Gram-negative bacteria. These factors explain the relatively high MIC values obtained, and the selective activity restricted to Gram-positive pathogens. This pattern aligned with the previous observations in *Ramalina farinacea*, whose extracts often inhibited only Gram-positive bacteria [48,52]. Methanolic extracts from several other lichens genera, including *Usnea* sp., *Everniastrum* sp., *Parmotrema* sp., *Ramalina* sp., *Cladonia* sp., *Heterodermia* sp., *Coccocarpia* sp., *Bulbothrix* sp., and *Peltigera* sp., also showed no activity against Gram-negative bacteria or *C. albicans* [45], suggesting that these variations in the results, not observed in other studies [37], may be due to the method used to determine the Minimum Inhibitory Concentrations and the sensitivity of the technique.

In Chile, lichen metabolites of pharmaceutical interest include usnic acid, atranorin, norstictic acid, gyrophoric acid, vicanicin, protolichesterinic acid, fumarprotocetraric acid, diffractaic acid, sphaerophorin, psoromic acid, variolaric acid, lobaric acid, salazinic acid, and physodic acid [53]. We are succesfully purified the major component calycin of *Chrysothrix* sp., a pulvinic acid derivative with known photoprotective properties due to its oxolane-carbonyl chromophore, which exhibits strong UV light absorption [54,55]. Although calycin has been previously reported in the lichen *Candelaria concolor* [55], no biological activity had been attributed to it. Our study is the first to demonstrate the antibacterial activity of calycin isolated from the Chilean lichen *Chrysothrix* sp.

The antibacterial mechanism of action of pulvinic acid derivatives (such as vulpinic and rhizocarpic acids) is primarily associated with the disruption of the plasma membrane and interference with cell division [56]. Given the chemical nature of calycin, a plausible mechanism is increased membrane permeability, as observed for other pulvinic acid derivatives. According to our findings, calycin’s efficacy appears confined almost exclusively to Gram-positive bacteria, likely because the outer lipopolysaccharide membrane of Gram-negative bacteria acts as a molecular barrier that prevents its penetration.

This membrane-targeting mechanism has been documented for pulvinic acid-rich extracts, which severely compromise membrane integrity in MRSA and other Gram-positive pathogens [57].

The shikimic acid pathway plays a central role in the biosynthesis of lichen secondary metabolites including pulvinic acid and pigments. The identification of calycin in this species reinforces the importance of this pathway and highlights its potential as a source of novel antimicrobial compounds.

## 5. Conclusions

This work presents the first integrated characterization of the chemical composition and antibacterial activity of the methanolic extract of *Chrysothrix* sp. from the Paposo Fog Oasis in northern Chile. The extract contained a complex mixture of secondary metabolites, from which calycin—a pulvinic acid derivative biosynthesized through the shikimic acid pathway—was identified as the major component. Both the crude extract and purified calycin exhibited selective inhibitory activity against Gram-positive multidrug-resistant pathogens, particularly *E. faecium* (MDR, VRE) and *S. aureus* (MDR, MRSA), while no significant activity was observed against Gram-negative bacteria. These findings highlight the relevance of *Chrysothrix* sp. as a source of bioactive metabolites and position calycin as a promising scaffold for antimicrobial development. Further studies should investigate its mechanism of action, cytotoxicity, and potential synergistic interactions with existing antibiotics to fully assess its therapeutic potential and pharmacological applicability.

## Figures and Tables

**Table 1:** In vitro antibacterial activity of methanolic extract and purified calycin from *Chrysothrix* sp., against *Staphylococcus aureus* and *Escherichia coli*, determined by disk diffusion assays.

Bacteria		Antibacterial Activity (mg/mL)			
		MeOH-Extract		Calycin	
	Profile	Concentration Tested	Inhibition Zone (Mean hd ± SDs)	Concentration Tested	Inhibition Zone (Mean hd ± SDs)
***S. aureus*** **ATCC 29213**	SUS, MSSA	0.25 0.5 1.0 2.0	0000	0.25 0.5 1.0 2.0	8.3 ^a^ 9.0 ^a^ 9.8 ^a^ 10 ^a^
**Ampicillin Positive control**			45 ± 1.4 ^b^		
**Methanol Negative control**			0 ^c^		
***E. coli*** **ATCC 25922**	SUS	0.25 0.5 1.0 2.0	0000	0.25 0.5 1.0 2.0	0000
**Ampicillin Positive control**			25 ± 0 ^b^		
**Methanol Negative control**			0 ^c^		

DR: Drug-Resistant; SUS: susceptible; Methicillin susceptible *S. aureus*; HD: inhibition halo diameter; SD: standard deviation. *p*-values were calculated using one-way ANOVA followed by Tukey HSD (α = 0.05). Superscript letters (a, b, c) indicate statistical significance (the distinct groups according to Tukey’s HSD test).

**Table 2:** In vitro activity of methanolic extract (ME) and calycin (PM) of *Chrysothrix* sp., against ESKAPE-E group by REMA.

ESKAPE-E Group	Profile	Antibacterial Activity (μg/mL)				Positive Control µg/mL
		MeOH-Extract (ME)		Calycin (PM)		
		MIC	MBC	MIC	MBC	
***E. faecium*** **ATCC 700221**	MDR, VRE	250	500	125	250	TET = 4
***S. aureus*** **ATCC 43300**	MDR, MRSA, VSSA	500	500	250	500	RIF = 1.28
***K. pneumoniae*** **ATCC 2146**	MDR, CBRE, ESβL (NDM-1).	>1000	>1000	ND	ND	COL = 8
***A. baumannii*** **ATCC BAA-1605**	XDR, CBRE	>1000	>1000	>1000	>1000	TET = 4
***P. aeruginosa*** **ATCC 35032**	MDR, CBRE	>1000	>1000	ND	ND	AMK = 4
***E. coli*** **ATCC 35218**	CBSU, βL (TEM-1)	>1000	>1000	ND	ND	AMK = 4

MeOH: Methanol; MDR: Multidrug-Resistant; VRE: Vancomycin-Resistant Enterococci; MRSA: Methicillin-Resistant *Staphylococcus aureus*; CBRE: Carbapenem-Resistant; CBSU: Carbapenem-Susceptible; ESβL: Extended Spectrum β-Lactamase; βL: β-Lactamase; TEM-1: Temoneira; NDM-1: New Delhi metallo β-Lactamase; XDR: eXtensively Drug-Resistant; TET: Tetracycline; COL: Colistin; AMK: Amikacin.

## Data Availability

The original contributions presented in the study are included in the article. Further inquiries can be directed to the corresponding author.

## Supplementary Materials

The following supporting information can be downloaded at: https://www.mdpi.com/article/10.3390/microorganisms14081766/s1, Table S1: Crystal data and structure refinement for Calycin, Table S2: Antimicrobial resistance profile of ESKAPE-E pathogens, Figure S1: Collection Area—Mirador Paposo.
