# Supplementary material for: Chrysothrix sp., a Lichen from Paposo Fog Oasis: Antibacterial Potential to Combat Multidrug-Resistant ESKAPE-E Pathogens
Source: Microorganisms. 2026 Aug 11;14(8):1766. doi: 10.3390/microorganisms14081766 (PMC13515766; doi:10.3390/microorganisms14081766)

Table S1. Crystal data and structure refinement for Calycin

Identification code	Calycin
Empirical formula	$C_{18}H_{10}O_5$
Formula weight	306.26
Temperature/K	295.15
Crystal system	Orthorhombic
Space group	$P2_12_12_1$
a/Å	6.757(5)
b/Å	12.995(9)
c/Å	15.153(10)
α/°	90
β/°	90
γ/°	90
Volume/Å^3	1330.5(16)
Z	4
ρ_{calc}g/cm^3	1.529
μ/mm^{-1}	0.113
F(000)	632.0
Crystal size/mm^3	0.02×0.01×0.06
Radiation	MoKα (λ = 0.71073)
2Θ range for data collection/°	5.376 to 53.022
Index ranges	$-8 \le h \le 8$, $-16 \le k \le 16$, $-19 \le l \le 18$
Reflections collected	33101
Independent reflections	2770 [R_{int} = 2.1726, R_{sigma} = 0.7871]

Data/restraints/parameters	2770/0/211
Goodness-of-fit on F^2	0.501
Final R indexes [I>=2σ (I)]	R_1 = 0.0574, wR_2 = 0.1264
Final R indexes [all data]	R_1 = 0.2893, wR_2 = 0.1853
Largest diff. peak/hole / e $Å^{-3}$	0.19/-0.21
Flack parameter	4(5)

Table S2. Antimicrobial resistance profile of ESKAPE-E pathogens.

**Acronym**	**Microorganism**	**Drug-resistant phenotype**	**Drug-resistance profile**
**E**	*E. faecium* ATCC 700221	MDR, VRE	AMP, CTX, CFT, CIP, CLI, CHL, ERY, STM, GEN, NOR, PEN, TEC, MET, VAN
**S**	*S. aureus* ATCC 43300	MDR, MRSA, VSSA	AMC, AMP, CLI, CRO, ERY, GEN, MET, OXA, PEN, SAM
**K**	*K. pneumoniae* ATCC 2146	MDR, CBRE, ESβL (NDM-1)	AMK, SAM, CFZ, FEP, CTX, CAZ, CRO, CIP, ETP, GEN, IPM, LVX, MEM, TZP, FOS
**A**	*A. baumannii* ATCC BAA-1605	XDR, CBRE	AMP, SAM, CFZ, FEP, CTX, CAZ, CRO, CIP, ETP, GEN, IMP, LVX, MEM, TZP, SXT, FOS
**P**	*P. aeruginosa* ATCC 35032	MDR, CBRE	AMP, SAM, CFZ, CTX, CRO, ERT, TGC, SXT
**-E**	*E. coli* ATCC 35218	CBSU, βL (TEM-1)	AMP, SAM

VRE: Vancomycin-Resistant Enterococci; VSE: Vancomycin-Susceptible Enterococci; MDR: Multi-Drug-Resistant; MRSA: Methicillin-Resistant *Staphylococcus aureus*; MSSA: Methicillin-Suceptible *Staphylococcus aureus*; VSSA: Vancomycin-Susceptible *Staphylococcus aureus*; VISA: Vancomycin-Intermediate *Staphylococcus* aureus; VRSA: Vancomycin-Resistant *Staphylococcus aureus*; CBSU: Carbapenem-Susceptible; CBRE: Carbapenem-Resistant; ESβL: Extended Spectrum beta-lactamase; βL: beta-Lactamase; NDM-1: New Delhi metallo β-lactamase; XDR: Extensively Drug-Resistant; VAN: Vancomycin; AMP: Ampicillin; DAP: Daptomicyn; OXA: Oxacillin, PEN: Penicillin; PIP: Piperacillin; MXF: Mocifloxacin; CRO: Ceftriaxone; FEP: Cefepime; CTX: Cefotaxime; SAM: Ampicillin/Sulbactam; CAZ: Ceftazidime; CFZ: Cefazoline; NIT: Nitrofurantoin; CIP: Ciprofloxacin; IPM: Imipenem; MEM: Meropenem; SXT: Trimethoprim/Sulfamethoxazole; TET: Tetracycline; CLI: Clindamycin; LZD: Linezolid; ERY: Erythromycin; TZP: Piperacillin/Tazobactam; GEN: Gentamicin; TOB: Tobramycin; AMC: Amoxicillin/Clavulanic acid; TIC: Ticarcillin/Clavulanic acid; LVX: Levofloxacin; AMK: Amikacin; MET:Methicillin; RIF: Rifampin; TGC: Tigecycline; CTT: Cefotetan; CFX: Cefuroxime; ETP: Ertapenem; AZT: Aztreonam; SNC: Synercid; COL:Colistin; FOS: Fosfomycin; CFT: Ceftaroline; CHL: Chloramphenicol; STM: Streptomycin; NOR: Norfloxacin.

Figure S1. Collection Area – Mirador Paposo

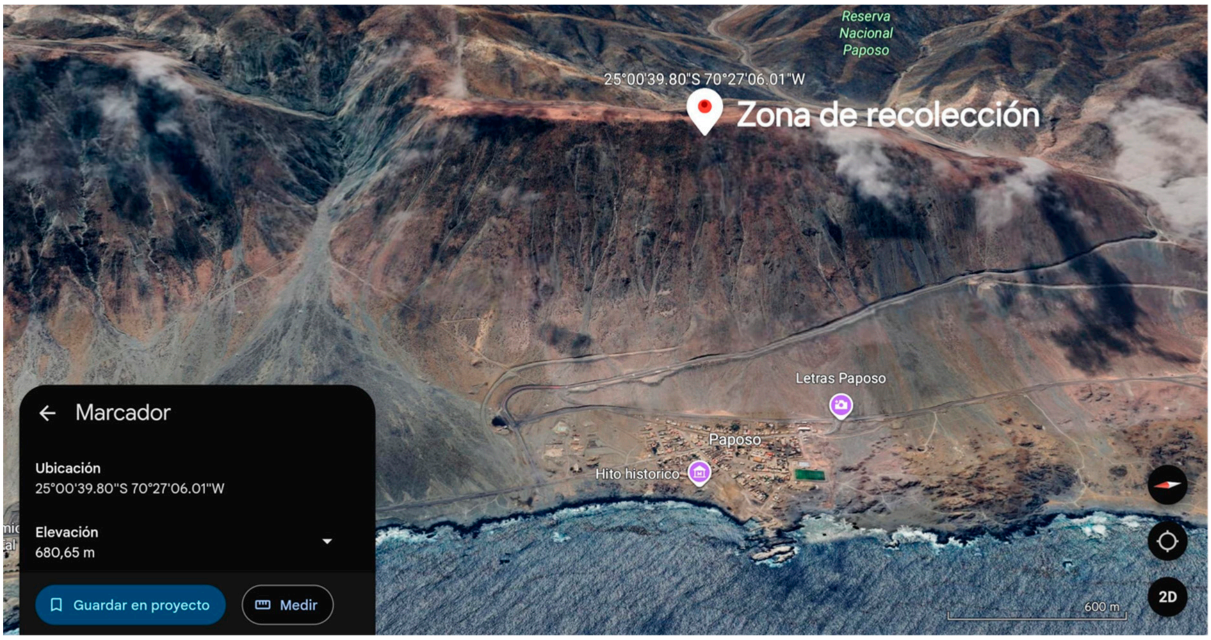

Supplement: Supplementary file 1 [file microorganisms-14-01766-s001.zip › microorganisms-4447851-supplementary.pdf]
